# Monkeypox Disease: An Emerging Public Health Concern in the Shadow of COVID-19 Pandemic: An Update

**DOI:** 10.3390/tropicalmed7100283

**Published:** 2022-10-03

**Authors:** Shamimul Hasan, Shazina Saeed

**Affiliations:** 1 Department of Oral Medicine and Radiology, Faculty of Dentistry, Jamia Millia Islamia, New Delhi 110025, India; 2 Amity Institute of Public Health, Amity University, Noida 201313, India

**Keywords:** monkeypox, poxvirus, public health menace, recent outbreak, skin rash, symptomatic therapy

## Abstract

The last few decades have witnessed an appalling rise in several emerging and re-emerging viral and zoonotic outbreaks. Such outbreaks are a lesson to learn from and seek insight into better disease monitoring and surveillance, thus preventing future outbreaks. Monkeypox, a viral zoonotic illness caused by the monkeypox virus, may no longer be endemic to the tropical rainforests of Central and West Africa. However, the current monkeypox outbreak in nonendemic countries is most likely due to failure to curb the disease dissemination in endemic African regions despite decades of constant outbreaks. The clinical manifestations are typified by a prodromal phase (fever, myalgia, malaise, and lymphadenopathy) followed by maculopapular or vesicular, or pustular cutaneous eruptions that eventually form encrustations and peel off. Children and the elderly, pregnant females, and individuals living with comorbidities (diabetes, HIV/AIDS, and lymphoproliferative ailments) are at a high risk of severe disease. Monkeypox is a self-limiting disorder, but its complications and pandemic potential signify its immense public health relevance. The recent ongoing monkeypox outbreak in nonendemic nations areas was identified with increased propensity in men who have sex with men (MSMs) with no travel history to endemic regions, emphasizing the changing trends in disease transmission. This review article provides an updated overview of the monkeypox disease taxonomy, pathogenesis, transmission, epidemiology, clinical and oral features, diagnostic aids, differential diagnosis, preventive aspects, and treatment protocol.

## 1. Introduction

Human monkeypox disease is a zoonotic disease due to the monkeypox virus. With the global smallpox eradication in 1980 and the concomitant cessation of the smallpox vaccination, monkeypox has recently emerged as a significant public health menace [1].

The monkeypox virus was first identified in Copenhagen, Denmark in 1958 during two flare-ups of a nonlethal skin disorder among imprisoned monkeys (Macaca fascicularis) that had been exported from Singapore for polio vaccine research [2,3]. The first human case was documented in an infant in Zaire (now the Democratic Republic of the Congo, DRC) in 1970 [4]. Between 1970 and 1980, 59 cases were documented in DRC. An active five-year surveillance period in DRC post-smallpox eradication in 1980 reported 338 cases [5]. However, since 2003, isolated cases have been noticed in nations outside of Africa, usually accompanied by a positive travel history to endemic regions [1,4]. The first monkeypox case outside Africa was documented in the Midwest of the United States (2003), when exotic Gambian giant rats from Ghana infected prairie dogs, eventually infecting 47 humans [3,6,7]. Historically, the largest monkeypox outbreak was reported in Nigeria in 2017, with a total of 197 suspected and 68 confirmed monkeypox cases [8]. The current multinational upsurge of monkeypox cases in nonendemic regions around the beginning of May 2022 demonstrated a shift in epidemiological patterns [9]. An upsurge was witnessed, particularly in gay and bisexual men, and men who have sex with men with no apparent travel history to the endemic regions [10].

Many animal species are prone to infection, and animal-to-human dissemination through handling and consuming infected animals is the predominant mode of transmission in endemic regions [11]. Transmission via droplet infection and physical contact with the skin rashes or scabs of infected individuals are the primary modes of transmission between humans, although the infection may also spread via fomites [12]. Vertical transmission from the mother to the fetus or newborn (congenital monkeypox) was also documented [13].

The current monkeypox outbreak documented a possible human-to-animal transmission. The first confirmed case of monkeypox virus infection in a dog was documented in Paris, that might have been acquired through human transmission [14].

However, the risk of monkeypox transmission following exposure in well-equipped healthcare settings is low, with only a single documented transmission event in the published literature [15].

The published literature demonstrates that the true reservoir host for the monkeypox virus is presently not known, although the virus may be naturally maintained in rodent populations and nonhuman primates [16].

The monkeypox virus has two different clades: the Congo basin clade and the West African clade. The West African clade exhibits less virulence, a mortality rate of <1%, and accounts for the recent monkeypox outbreak [17]. The disease clinical array varies from mild to severe and lethal. Typically, a 1–4 day prodromal phase of fever, malaise, fatigue, and headache is followed by centrifugal development of well-defined macular–papular, vesicular, and pustular eruptions, which eventually encrust and shed off. Early onset lymphadenopathy, usually coinciding with the fever onset, is the hallmark feature [18]. Pneumonitis, encephalitis, vision-threatening keratitis, and secondary bacterial infections account for the commonly encountered complications [6].

Most recent infections have milder and atypical clinical manifestations, thus delaying the diagnosis and spread of infection. Although most cases exhibit a skin rash, it may be confined to the genital, perigenital, and perianal regions. Additionally, prodromal symptoms of fever, malaise, and myalgia may not be seen in all patients. These features delineate the current outbreak from classic monkeypox cases, thus posing a diagnostic threat and delaying the rapid isolation of affected patients [10].

The recent human monkeypox outbreak indicates changes in either the virus’ biologic features or human practices, or both. These changes may be attributed to the fading smallpox immunity, the diminution of coronavirus disease 2019 (COVID-19) preventive strategies, resumed international travel, and sexual intimacy related to mass congregations [19].

This recent monkeypox outbreak in many nations across nonendemic countries has also emphasized the lack of knowledge regarding the risk of infection transmission in healthcare settings [15].

It is imperative to assess the knowledge of medical students about emerging viral infections. This is instrumental in preparing them as future healthcare workers and would also motivate them to work during infectious disease outbreaks. Moreover, delineating the association between disease knowledge and attitude towards the prevailing conspiracy theories may influence the understanding of various health-seeking behaviors (including the increased adherence to preventive strategies such as vaccination) [20].

Awareness regarding the risk of transmission and various healthcare exposures attributed to higher-risk groups is of paramount importance not only for implementing infection control and preventive measures, but also to appraise guidelines for postexposure surveillance and prophylaxis [15].

Recently published studies have revealed a lack of knowledge, practice, and attitudes regarding monkeypox among physicians, medical students, and the general population. A recent World Health Organization (WHO) report suggested that a lack of knowledge regarding the monkeypox infection served as a major obstacle in averting the re-emergence of the current outbreak. The recent upsurge in human monkeypox incidence highlights the significance of early screening and detection, and the implementation of various preventive strategies [8,20].

Enhancing knowledge may be regarded as the initial step in modifying attitudes and behaviors [20]. Hence, the general population needs to be appraised not only about the disease and its complications, but also about adopting the various preventive strategies, thus curbing the global dissemination of the monkeypox virus [8]. It is also imperative to practice precautionary protective measures that diminish zoonotic and human-to-human transmissions [21].

Currently, there is no definitive treatment regimen for monkeypox virus infection, and supportive care and symptomatic management form the mainstay of treatment [22].

The recent monkeypox outbreak was declared a public health emergency of international concern (PHEIC) by the World Health Organization on 23 July 2022 [23]. Public health strategies have not been effective in limiting monkeypox dissemination. The atypical presentations in the current outbreak also pose a challenge for contact tracing [24].

In the past 20 years, India has witnessed several emerging and re-emerging viral and zoonotic diseases [25]. The global climatic changes predispose to a higher risk of cross-species dissemination of many viral and zoonotic ailments [26].

## 2. Taxonomy

Human monkeypox disease is caused by the monkeypox virus belonging to the Poxviridae family [1]. The family is subdivided into two subfamilies: Chordopoxvirinae and Entomopoxvirinae. The Chordopoxvirinae family primarily infects the vertebrates and is subclassified into 18 genera, including Orthopoxvirus. The Entomopoxvirinae subfamily infects invertebrates and is subclassified into four genera (Alphaentomopoxvirus, Betaentomopoxvirus, Gammaentomopoxvirus, and Deltaentomopoxvirus) [3].

The Orthopoxvirus (OPXV) genus has more than 10 member species, including the variola (smallpox) virus (VARV), vaccinia (the smallpox vaccine) virus (VACV), camelpox virus (CMLV), cowpox virus (CPXV), and several unique species isolated from infected humans or primates [2].

## 3. Transmission

Monkeypox infection primarily spreads via zoonotic and human–human transmission, usually through direct physical contact, a scratch or bite from an infected animal, or consumption of the host animal. Individuals with no smallpox immunization sleeping on the floor in recently deforested regions, and handling and consuming dead animals are more vulnerable to the zoonotic spread of the infection [12]. Respiratory droplet infection and direct contact with skin exanthem or scab lesions continue to be the principal modes of transmission between humans. Transmission may also occur by touching inanimate objects (clothing, beddings, etc.) contaminated with the virus, and the splashing or aerosolization of virus-containing particles when inappropriate biosafety procedures are undertaken [27].

Several monkeypox cases have been reported in men having sex with men (MSM). Some of these cases had documented international travel to nonendemic countries and attending mass gathering events or festivals, such as Maspalomas (Gran Canaria) 2022 Pride, thus suggesting a possible role of sexual intercourse that may aid in the transmission of the disease [28,29]. A recent study reported that the monkeypox virus detected in a patient’s semen specimens during the acute infection phase might contain a replication-competent virus. The cytopathic effects observed after viral inoculation in the cell growth medium illustrate viral replication competency. Moreover, the authors proposed that monkeypox might have a genital reservoir as prolonged seminal viral shedding occurs, even at low viral copies [30].

**Figure 1:**
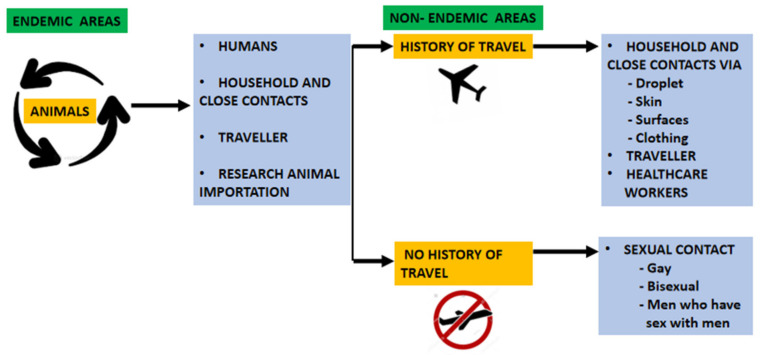
Depicts various modes of transmission for monkeypox infection.

Transmission can also occur via the fetus–maternal route (congenital monkeypox) or during close contact during and after birth [3,7,26]. Figure 1 depicts various modes of transmission for monkeypox infection.

## 4. Epidemiology

Historically, the monkeypox virus was first isolated in Copenhagen, Denmark in 1958 among imprisoned cynomolgus macaques imported from Singapore for research activities [2,31].

The first human monkeypox case was reported in 1970 in the Democratic Republic of Congo (DRC) when the region was thought to be variola-free and was under surveillance for smallpoxlike illnesses [2,4,7]. Since that time, DRC has been an endemic monkeypox region, although the virus continues to spread to other African countries, primarily in Central and West Africa [3,4].

Monkeypox cases were limited to the tropical rain forests of West and Central Africa, primarily due to close interaction with infected animals due to mass deforestation, the progressive fading of smallpox immunization, and ameliorated disease surveillance and diagnostic facilities in the African region [32].

Between 1970 and 1980, 59 cases were reported. A five-year period of active disease surveillance in DRC post smallpox eradication identified 338 cases [5]. However, the recent re-emergence outside of Africa poses the risk of identifying new risk factors. The first monkeypox case outside Africa was reported in the Midwest of the United States (2003), when exotic Gambian giant rats from Ghana infected prairie dogs, eventually infecting 47 human beings [3,6,7]. A monkeypox outbreak in Sudan accounted for a total of 10 confirmed and 9 suspected monkeypox cases from September to December 2005 [33]. In 2017, 122 documented cases in Nigeria showed both zoonotic and human–human modes of transmission [3,5].

Several isolated cases of monkeypox disease were documented in many non-African countries from 2018 to 2021, but they all shared a commonality in the history of travel to Nigeria: the United Kingdom reported 7 cases, Israel and Singapore reported 1 case each, and 2 cases were documented in the U.S [2,34]. In all the cases except one reported in the UK, human-to-human transmission was not detected [2,35].

Before the recent emergence of monkeypox disease in May 2022, epidemiological trends depicted that the disease occurred in individuals either with a travel history to endemic regions of Africa or those exposed to infected animals. However, in mid-May 2022, World Health Organization documented 257 laboratory-confirmed cases and approximately 120 suspected monkeypox cases in 23 nonendemic nations [36].

The recent monkeypox outbreak had an upsurge, particularly in men who have sex with men with no apparent travel history to the endemic regions [2,37,38].

By 22 September 2022, a total of 64,916 confirmed monkeypox cases were reported to WHO from 106 countries, of which 64,336 cases were documented in countries that have not historically reported monkeypox, and 580 cases in countries that historically reported monkeypox. Monkeypox cases were seen in 99 countries that have not historically reported monkeypox, and in 7 countries that have [39].

**Figure 2:**
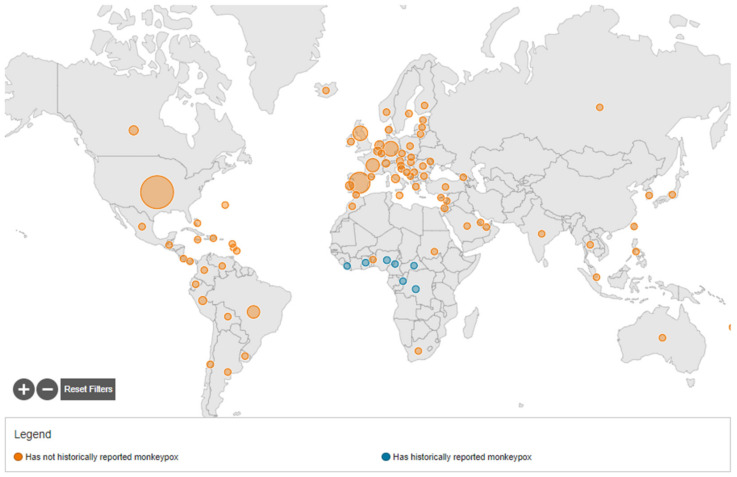
Describes the geographical distribution of monkeypox virus infection.

On 15 July 2022, the first case of monkeypox in the WHO Southeast Asia Region was documented in India, in a 35-year-old man who returned from the Middle East [40]. By 22 September 2022, 12 cases of monkeypox and 1 death were reported in India [31]. Figure 2 describes the geographical distribution of monkeypox virus infection.

## 5. Pathogenesis

MPXV can invade human cells by the following modes: (a) direct integration between the molecules on the viral coat and the receptors on the human cell membrane [3,41]. Four viral proteins can facilitate the integration: D8 binds to chondroitin sulfate, A27 and H3 proteins bind to heparan sulfate, and the A26 protein binds to laminin [42]. The process of attachment is followed by the rapid dispersal of the viral envelope in the host cell membrane. Viral proteins and enzymatic factors are released in the cytoplasm, thus weakening the host defense and inducing the expression of early genes. Later, the formation of early proteins, DNA, and synthesis of intermediate transcription factors occur, with the (b) endosomal uptake through a macropinocytosis process involving actin [3,41].

Eleven proteins mediate the viral entry into the host cell. Nine proteins (A16, A21, A28, G3, G9, H2, J5, L5, and O3) are integral components of the entry fusion complex (EFC), whereas the other two proteins (F9 and L1) are EFC-associated [42]. Once it has gained access to the host cell, the monkeypox virus replicates at the site of inoculation before dispersing to the regional lymph nodes [43]. The monkeypox virus generates sufficient proteins for both transcription and replication, thus replicating within the infected host cell cytoplasm rather than in the nucleus, a contrasting feature with many DNA viruses [44].

**Figure 3:**
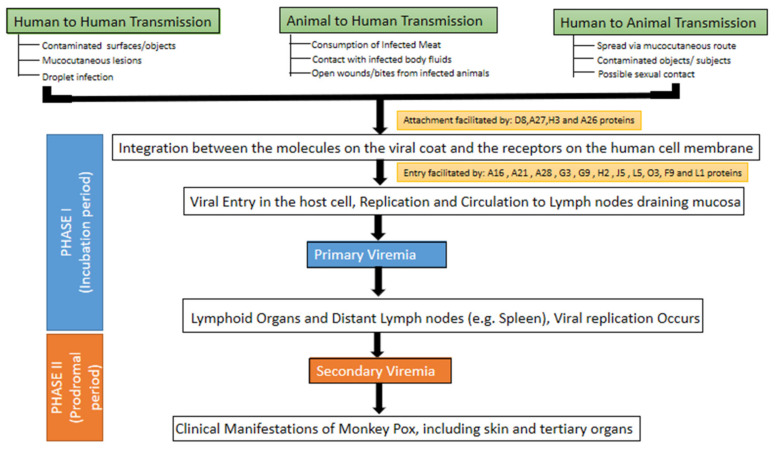
Pathogenesis of monkeypox virus infection.

This is followed by primary viremia causing the viral dissemination to other sites. This phase depicts the incubation period and ranges from 1–2 weeks to a maximum of 3 weeks. Prodromal symptoms such as fever and lymphadenopathy typically correspond with a 1–2 day phase of secondary viremia. Infected patients may be infectious during the secondary viremia phase. Lesions commence in the oropharynx, followed by skin eruptions (Figure 3) [43,45]. 

## 6. Clinical Features

The incubation period of monkeypox varies in the range of 5–24 days, with a mean of 12 days [18,22]. Monkeypox virus infection typically describes a biphasic clinical presentation. A prodromal period of fever, headache, malaise, myalgia, and lymphadenopathy usually precedes the appearance of cutaneous rashes 2–4 days later [3,12,17,22,46].

Cutaneous lesions exhibit a salient pattern of progression, commencing as a well-defined exanthema that advances through macular, papular, vesicular, and pustular stages in a distinctive centrifugal pattern [2,6,7,17,27,44,46]. A typical rash lesion is vesicopustular. The lesions progress into papules by the third day, and vesicles (raised and fluid-filled) by the fourth to fifth day. By the sixth to seventh day, the lesions become firm, deep-seated pustules (sharply raised and filled with opaque fluid) that may umbilicate or coalesce. They eventually dry up and exhibit encrustations by the end of the second week, and the scabs exfoliate after a week. The lesions heal with hyperpigmented or hypopigmented atrophic scars, and patchy alopecia. Facial muscle contracture or deformity following healing of ulcerated facial lesions may also occur [13].

Lesions exhibit a site predilection, primarily affecting the face (95% of cases), followed by the palms and soles (75% of cases), and mucous membranes (70% of cases). The skin lesions infrequently affect the trunk and genitals [2,3,7,26,46]. On the basis of the number of lesions, the exanthem may be classified as mild (<25 skin lesions), moderate (25–99 skin lesions), severe (100–250 skin lesions), and very severe (>250 skin lesions) [47]. Scalp lesions were also reported in a few patients [6].

Severe monkeypox infections may present as encephalitis, pneumonia, secondary bacterial skin infection, and ocular diseases leading to keratitis, blurred vision, and corneal scarring, although most infections are relatively milder and self-limiting within 2–4 weeks. Neonates, children, and individuals with comorbidity and immunodeficiency are the most susceptible groups to infection [3,5,6,7,26,48,49]. Gastrointestinal symptoms such as vomiting and diarrhea may result in severe dehydration in an infected person. The dehydration may be further worsened with associated mouth and throat ulcers that may pose difficulties with maintaining nutrition. Sepsis and septic shock may primarily occur due to overly exaggerated immune responses [50]. Individuals coinfected with influenza may also present with bronchopneumonia [22,48].

There is a preliminary affirmation regarding a spectrum of neurological and psychiatric monkeypox presentations ranging from nonspecific neurological manifestations (such as headache and myalgia) to infrequent but more lethal neurological complications (such as seizures and encephalitis). However, there is a lack of conclusive evidence on the psychiatric sequelae and monkeypox-related nervous system presentations that may warrant surveillance within the current MPX outbreak [51].

The recent 2022 monkeypox upsurge has shown atypical symptoms in several cases. For instance, although the typical rash is still visible, it is usually limited to the genital, perigenital, and perianal sites, and displays different developmental stages. Additionally, patients may exhibit only mild or absent prodromal features, thus hindering the diagnosis and speedy quarantine of the patient. Therefore, it is imperative to encompass a wide array of disease manifestations for early and accurate diagnosis [10,46].

A recently published case series on 23 patients with a history of clear exposure during the current outbreak revealed that 95% of the patients reported a rash lesion (around two-thirds of the cases had <10 skin lesions). The frequency of the reported lesions was as follows: anogenital lesions (73%), mucosal lesions (41%), and a single genital ulcer (10%). The anorectal lesions caused severe anorectal pain in 11.5% of the patients, a feature not documented previously. Multiple types of lesions at the same time were also reported in the patients in the current outbreak, another distinguishing feature from the previous monkeypox outbreaks [19].

## 7. Orofacial Features

Dental professionals should remain watchful to the initial signs of a facial exanthem. Scrupulous steps should be taken to augment the knowledge among dental professionals regarding the presenting manifestations. As monkeypox infection may spread via short-range aerosol, dental professionals are particularly at a higher risk due to a large amount of aerosol-generating activity in dentistry [52]. Oral involvement in the form of a sore throat, oral ulcers, and dysphagia was reported by Gregory et al. [53]. Patel et al. reported oropharyngeal lesions, tonsillar erythema, pustules, edema, or abscess [7]. Oropharyngeal symptoms, such as pharyngitis, epiglottitis, odynophagia, and oral or tonsillar lesions were reported as the initial lesions in a few patients [19]. Rashes may be seen in the oral cavity, deteriorating the nutrition [17]. The occurrence of oral and throat ulcers, nausea, vomiting, and cervical lymphadenopathy during the early course of the illness may lead to a decreased appetite [48].

## 8. Diagnosis

Human monkeypox infection is usually diagnosed clinically with the characteristic skin exanthem. A comprehensive clinical history, including travel to endemic regions, occupation and contact with infected animals, and a confirmed laboratory diagnosis are imperative for differentiating the various rash-associated illnesses [44].

It is imperative to use personal protective equipment (PPE) during specimen collection. The specimen should be obtained from two distinct appearing lesions on distant body sites using sterile, dry synthetic swabs with plastic, wood, or a thin aluminum shaft (not cotton swabs). A sufficient amount of viral DNA may be obtained with vigorous swabbing, and unroofing the specimens may not be necessary nor recommended because of infection control or sharps injury concerns [54].

The polymerase chain reaction (PCR) test is the gold standard for a confirmed diagnosis of a suspected case. Certain real-time PCR assays can differentiate both the monkeypox virus from other orthopoxviruses and between two monkeypox clades [51]. Certain guidelines have been issued by the Government of India for the diagnosis of monkeypox infection. Samples including skin scrapings, EDTA blood, nasopharyngeal/oropharyngeal swab, and serum urine are processed for Orthopox genus-specific PCR. If positive, the specimens are further processed for monkeypox-specific PCR [55].

In cases where monkeypox infection is still suspected despite a negative PCR test result, other tests, as mentioned in Table 1, can be employed [3,27,44,46].

Biosafety Level 3 (BSL-3) containment laboratories should be used during the handling of any questionable infectious specimens. Due to its precision and credibility, GeneXpert was advocated as a potential diagnostic platform that may expand and hasten current MPXV detection abilities in endemic regions [44]. The specific benefits of GeneXpert and other similar point-of-care PCR platforms (e.g., TaqMans-MGB real-time PCR) may be more related to expanding the potential accessibility of testing and decreasing turnaround time.

## 9. Differential Diagnosis

As the clinical presentation of monkeypox closely simulates that of smallpox and chickenpox, a definitive diagnosis is imperative to keep the natural disease in check or for the initial identification of an impending bioterrorism outcome [56]. Smallpox has been wiped out since 1980, and the Centers for Disease Control and Prevention (CDC) in the United States, and the Russian State Centre for Research on Virology and Biotechnology in the Russian Federation are the two laboratories where it is maintained. However, bioterrorism related to its spontaneous release is a possible but exceedingly rare event [57].

Many symptoms of monkeypox mimic those of smallpox. However, a relatively milder infection manifesting as early onset lymphadenopathy and often coinciding with the fever onset is the hallmark feature of monkeypox [2,3,17,22,26,38,53]. Firm and mildly tender 1–4 cm-sized maxillary, cervical, and inguinal lymphadenopathy is usually seen in unvaccinated patients (84% of cases), and vaccinated patients (54% of cases) [3,5,17]. Early onset lymphadenopathy demonstrates a more vigorous immune response and detection of MPXV than those for variola [3,58].

Up to 50% of suspected MPX cases in endemic regions may be confused with chickenpox [22]. A key distinctive manifestation of monkeypox and smallpox is that the skin exanthema on the body is all in the same stage of development [2,58]. However, the 2022 monkeypox outbreak may display lesions in different developmental stages [10,46].

The differentiating features among monkeypox, smallpox, and chickenpox are summarized in Table 2 [10,26,46,56,59]. Clinical symptoms may aid in differentiating monkeypox virus infections from other causes of vesiculopustular eruptions, but a definitive diagnosis always entails laboratory corroboration [56].

## 10. Prevention

Despite ongoing attempts for the development of various therapeutic modalities, basic public health interventions such as immediate case isolation, contact tracing, avoiding contact with infected animals or materials, the use of personal protective equipment, and practicing good hand hygiene are effective means to control the spread of human monkeypox [44]. Community engagement in the form of health education, health awareness, advocacy, and large vaccine drives are the mainstays of prevention [60].

Hospitalized individuals do not require combined airborne-droplet and contact isolation precautions, and negative pressure rooms are not needed [61].

Healthcare workers should practice a strict mask policy by wearing well-fitted N95 masks, gloves, and personal protective equipment before any contact with a suspected case [3].

Emphasizing public awareness and educational interventions via appropriate health advocacy are simple yet effective measures that are beneficial in reducing the transmission of the virus [17]. Increasing the awareness of the general population about the various risk factors and educating them to adopt the preventive measures are the key preventive protocol for monkeypox. Healthcare workers and household members are the potential risk groups. It is imperative for healthcare workers in contact with confirmed or suspected monkeypox cases, or those handling their samples to strictly follow universal infection control measures. If possible, previously vaccinated individuals against smallpox should be selected to care for the patients [60].

Men who have sex with men are advised to limit the number of sexual partners, avoid sex with new partners, and share contact details with any new partners to facilitate contact tracing if required. Stigma and prejudice can be as threatening as any virus and can augment the outbreak. As we have seen with COVID-19, there is a rapid online spread of rumors and misinformation. Hence, healthcare organizations can collaborate with social media platforms, tech companies, and news organizations to avert and combat the spread of misleading information [62].

The published literature has documented that smallpox vaccination efficaciously averts other Orthopoxvirus infections, including monkeypox. When given during the initial incubation phase, it may impede the disease onset or dilute the ill effects of the disease and its outcomes. However, immunocompromised patients are still at a higher risk of severe adverse events. Smallpox eradication in 1980 resulted in vaccination cessation against the viral illness, predisposing individuals to a risk for acquiring monkeypox disease. ACAM2000 (live vaccinia virus), LC16 m8 (attenuated vaccinia virus), and modified vaccinia Ankara (attenuated vaccinia virus) is the next-generation smallpox vaccines that not only provide enhanced protection but also induce antibody development in atopic and immunosuppressed individuals [3,43].

ACAM2000 is a replication-competent vaccinia virus vaccine that had been employed for manufacturing the Dryvax vaccine, one of the vaccines used for smallpox eradication. Replication-competent poxvirus strains can result in human clinical infection by producing an infectious virus that can be transmitted to others. Being a replication-competent strain, ACAM2000 is associated with serious adverse events (e.g., progressive vaccinia, eczema vaccinatum, and myopericarditis). ACAM2000 was the only Orthopoxvirus vaccine licensed by the Food and Drug Administration (FDA) during 2015–2019 [63].

Replication-deficient poxvirus strains (e.g., modified vaccinia Ankara (MVA), ALVAC, and TROVAC) do not cause human clinical infection and do not produce infectious viruses in humans. Replication-deficient poxvirus strains are associated with a considerably lower risk of adverse events compared with replication-competent strains. In 2019, FDA licensed JYNNEOS, a replication-deficient MVA vaccine, for smallpox or monkeypox disease prevention in individuals aged ≥18 years who are more prone to infection. In November 2021, the Advisory Committee for Immunization Practices (ACIP) unanimously opted for JYNNEOS as an alternative to ACAM2000 for primary vaccination and booster doses [63].

JYNNEOS involves two vaccine doses given at an interval of 28 days. However, vaccine protection is not accorded until 2 weeks after receiving the second dose. ACAM2000 involves 1 vaccine dose, and peak vaccine protection is accorded within 28 days [51].

However, a recently published case documented that a previously healthy 34-year-old male contracted monkeypox infection, despite being immunized against smallpox with ACAM2000 vaccine 8 years back. He had had unprotected anal sex with a men 11 days earlier and then presented constitutional symptoms preceding the painless penile lesions that later coalesced. This case highlights the fact that, although vaccination is a key preventive measure, it alone does not ensure immunity from monkeypox. The vaccine should supplement and not replace public health strategies that intend to diminish high-risk health behaviors [64].

## 11. Treatment

Currently, there is no conclusive treatment protocol for monkeypox virus infection. Symptomatic and supportive management is the cornerstone of treatment. This includes maintaining nutrition, fluid and electrolyte balance, symptomatic management with antipyretics or analgesics, the timely diagnosis of secondary infections, and efficient management with suitable antimicrobials [65]. Table 3 summarizes the supportive treatment of monkeypox [55].

As the majority of the cases are self-limiting, no specific antivirals are needed [13]. However, patients with severe complications, pregnant females, patients in the pediatric and geriatric age group, and immunosuppressed patients warrant definitive antiviral therapy [3,13,43,46]. Table 4 summarizes the treatment of complications and severe forms [47].

No US Food and Drug Administration (FDA)-accepted remedies exist for monkeypox. Cidofovir, brincidofovir, and tecovirimat are the few antiviral drugs that may have a potent action against the monkeypox virus [2,3,17,26,46,49]. Cidofovir exhibits an intro action against pox viruses and inhibits viral polymerase enzyme activity. However, it is extremely nephrotoxic. Brincidofovir (CMX-001), a prodrug of cidofovir, is associated with lesser nephrotoxic effects, but no conclusive advantage was documented with its use in three monkeypox cases in the UK. Recently, tecovirimat has been authorized for the management of Orthopoxvirus infections (smallpox, monkeypox, cowpox, and vaccinia) in the USA, Canada, and Europe. It inhibits the function of a viral envelope protein (VP37), which blocks the viral maturation and dispersal from the infected cell, thus, preventing the viral spread within the infected host. It is also efficacious in protecting animals from rabbitpox and monkeypox with no serious adverse events [13,51].

Additionally, vaccinia-vaccination-related complications may be managed by FDA-approved vaccinia immune globulin intravenous (VIGIV) [2,3,17,26,46,49].

## 12. Conclusions

The recent COVID-19 and monkeypox outbreaks have emerged as a serious threat to healthcare. Progressively fading immunity related to the cessation of smallpox vaccination, mass deforestation leading to close human interaction with wild animals, travel to endemic regions, and change in sexual practices may be some of the proposed reasons for the global dissemination of monkeypox illness. International collaboration for enhanced monitoring and identification of monkeypox cases are indispensable mechanisms for comprehending the constantly changing epidemiologic pattern of monkeypox disease.

## Figures and Tables

**Table 1:** Delineates the various diagnostic aids for monkeypox virus infection.

Diagnostic Tests	Description	Specimen Used
Polymerase chain reaction (PCR)	It is based on the nucleic acid amplification test (NAAT) that demonstrates monkeypox DNA (deoxyribonucleic acid); real-time PCR is currently the gold standard.	Lesion exudate/crust sample
Viral culture	The virus is cultured and isolated from a patient specimen.	Lesion exudate
Electron microscopy	Morphologically demonstrate the pox viruses.	Biopsy, scab lesion, vesicular exudate
Immunohistochemistry	Demonstrates Orthopoxvirus-specific antigens.	Biopsy
Anti-Orthopoxvirus immunoglobulin G (IgG) and immunoglobulin M (IgM) tests	Evaluate a recent or previous exposure to Orthopoxvirus.	Blood sample

**Table 2:** Represents the distinguishing features of common rash-associated viral illnesses.

Disease Features	Monkeypox	Smallpox	Chickenpox
HISTORY			
Mode of infection	Previous outbreaks- (a) Zoonotic-direct physical contact; scratch/bite of an infected animal; consumption of host animal (b) human–human respiratory droplet infection; contact with skin exanthem/virus contaminated inanimate objects; vertical transmission	Respiratory droplet infection and by contact with skin exanthem	Airborne disease, spread by coughing/sneezing, and by contact with skin exanthem
	2022 Outbreak Possible sexual route as this outbreak witnessed cases, particularly in gay and bisexual men, and men who have sex with men		
INCUBATION PERIOD			
	7 to 17 days	7 to 17 days	10 to 21 days
PRODROMAL PHASE (mild or absent in the 2022 monkeypox outbreak)			
	Yes (1–4 days)	Yes (2–4 days)	Yes (0–2 days)
CLINICAL EVALUATION			
Fever	1–3 days before rash	2–4 days before rash	1–2 days before rash
	Moderate fever 38.5–40.5 °C	High fever, often more than 40 °C	Mild/no fever Usually less than 38.8 °C
Malaise	Moderate	Moderate	Mild
Lymphadenopathy	Moderate	Absent	Absent
Headache	Moderate	Severe	Mild
CUTANEOUS EXANTHEM			
Rash development	Slow	Rapid	Rapid
Appearance	Umbilicated	Umbilicated	Dewdrop
Pattern	Centrifugal	Centrifugal	Centripetal
Evolution	Previous monkeypox outbreaks: monomorphic (single stage of development)	Monomorphic	Pleomorphic
	2022 monkeypox outbreak: pleomorphic (different stage of development)		
Distribution	Previous monkeypox outbreaks: denser on the face, palms and soles, and mucous membranes	Starts on the face, followed by spreading to arms and legs, and hands and feet.	Starts on the chest, back, and face, and then spread over the entire body, including inside the mouth, eyelids, or genital area
	2022 monkeypox outbreak: usually limited to the genital, perigenital, and perianal sites, with mild or absent prodromal features		
Depth (mm)	Superficial–deep (2–6 mm)	Deep (4–6 mm)	Superficial (2–4 mm)
Peeling/Desquamation of rash	22–24 day	14–21 days	6–14 days
COMPLICATIONS			
Encephalitis	Less than 1%	Less than 1%	Less than 1%
Pneumonitis	Yes up to 12%	Possible	Yes up to 16%
Eye complications	Yes up to 5%	Yes up to 9%	None
Secondary Soft tissue infections	Yes	Yes	Yes

**Table 3:** Symptomatic and supportive treatment for monkeypox.

Component of Management	Signs/Symptoms	Treatment Protocol
Protection of compromised skin and mucous membranes	Skin exanthem	Washing with antiseptic Mupironic Acid/fucidin Light dressing in cases of extensive lesion present Not touching or scratching lesions Antimicrobials in cases of secondary infections
	Genital lesions	Sitz Bath
	Conjunctivitis	Usually, self-limiting Ophthalmologist consultation in cases of persistent symptoms, or pain or visual disturbances
Symptomatic relief	Fever	Tepid sponging Antipyretics (paracetamol) as needed
	Itching/Pruritus	Topical Calamine lotion Antihistamines
	Nausea and vomiting	antiemetics
	Headache/malaise	Paracetamol and adequate hydration
Nutritional support, fluid and electrolytes balance	Dehydration can occur in association with poor appetite, nausea, vomiting, and diarrhoea	Encouraged nutritious and balanced diet Encouraged oral rehydration solution (ORS) or oral fluids Intravenous fluids if required

**Table 4:** Management of complications and severe monkeypox forms.

Complication	Management
Skin exfoliation	Patients with heavy rash load may develop skin exfoliation that can be critical, leading to dehydration and protein loss. Evaluation of affected skin percentage and consider a treatment such as burns. Ensuring adequate hydration and nutrition. Consultation with surgeons, dermatologists, and/or wound care specialists, as severe cases may require skin grafting. Bedside or surgical debridement as needed.
Necrotizing soft tissue infection	Should be suspected if a patient develops oedema, crepitus, foul discharge, or pain out of proportion to the appearance of infection. Though it may be seen in monkeypox infection, bacterial pathogens should also be considered Broad-spectrum antibiotics with staphylococcus sp. and streptococcus sp coverage. Surgical debridement
Ocular lesions	Eye care with ophthalmologist consultation (eye lubrication and protective eye pads) Ophthalmic antibiotics or antivirals if indicated for coinfection. Trifluridine eye drops (sometimes used for other orthopoxviruses or herpetic eye infections) may hasten healing and avert long term damage from scarring Vitamin A supplementation, especially for malnourished children Avoid steroid ointments (may prolong the presence of MPX in ocular tissue)
Acute respiratory distress syndrome	Oxygen, noninvasive ventilation, mechanical ventilation.
Severe dehydration and hypovolaemic shock	Resuscitation with intravenous or intraosseous (IV/IO) fluid and close observation of fluid responsiveness.
Sepsis and septic shock	Early identification, and management of infection with supportive care (fluid resuscitation to maintain organ perfusion, and to reduce and prevent further organ injury)
Encephalitis	Lumbar puncture for cerebrospinal fluid (CSF) evaluation Monitor and evaluate airway, breathing, circulation, and disability (ABCD) and provide emergency treatments. Monitor neurological status Control seizures with antiepileptics. Antibiotics or antivirals if indicated for coinfections.
Nutritional considerations	Limited food intake due to weakness, assisted feeding by a health care provider may help. Enteral nutrition in cases of compromised oral intake Nasogastric feeding with a nasogastric tube (ensure proper tube placement to avoid aspiration risk) Special care with critically unwell patients, reduced food intake for >5 days, low BMI, a history of alcohol abuse/drugs (chemotherapeutics, insulin, antacids, or diuretics) and consider slow enteral feeding with close monitoring.

## Data Availability

Not applicable.
